# Détection du virus de la fièvre de la vallée du Rift chez *Culex pipiens* à Tahoua au Niger

**DOI:** 10.48327/mtsi.v4i2.2024.512

**Published:** 2024-04-30

**Authors:** Souleymane MAHAMANE IRO, Adamou LAGARE, Abdoul-Aziz MAIGA, Zara NOUHOU, Haladou GAGARA, Hadiza OUSMANE, Abdoul-Nasser HASSOUMI SANDA, Halima ZAMANKA, Soumana AMADOU, Fouta BOUBAKAR, Ibrahima ISSA ARZIKA, Laminou IBRAHIM MAMAN

**Affiliations:** 1 Centre de recherche médicale et sanitaire (CERMES), Niamey, Niger; 2 Laboratoire d’entomologie fondamentale et appliquée, Université Joseph KI-ZERBO (UJKZ), Ouagadougou, Burkina Faso; 3 Faculté des sciences agronomiques-Université Abdou Moumouni (UAM) de Niamey, Niamey, Niger; 4 Laboratoire central de l’élevage de Niamey (LABOCEL), Niamey, Niger; 5 Faculté des sciences et techniques, Université Abdou Moumouni (UAM) de Niamey, Niger; 6 École nationale de santé publique de Zinder (ENSP), Zinder, Niger

**Keywords:** Fièvre de la vallée du Rift, FVR, Virus, *Culex pipiens*, Épicentre, Tahoua, Tassara, Tchintabaraden, Abalak, Agadez, Ingall, Niger, Afrique subsaharienne, Rift Valley Fever, RVF, Virus, Culex pipiens, Epicenter, Tahoua, Tassara, Tchintabaraden, Abalak, Agadez, Ingall, Niger, Sub-Saharan Africa

## Abstract

**Introduction:**

La fièvre de la vallée du Rift (FVR) est une arbovirose responsable de fréquentes épizooties et épidémies en Afrique subsaharienne et dans la péninsule arabique. En 2016, le Niger a enregistré le premier foyer de FVR dans la région de Tahoua avec des conséquences importantes sur la santé animale et humaine. Le but de cette étude est de rechercher la présence du virus de la FVR (VFVR) chez les vecteurs potentiels de la maladie.

**Matériels et méthodes:**

Il s’agit d’une enquête transversale sur les vecteurs potentiels du VFVR conduite dans les régions de Tahoua et d’Agadez en août 2021. La capture des moustiques a été réalisée à travers les méthodes par pulvérisation et pièges lumineux CDC. Après identification morphologique, l’ARN viral a été extrait en utilisant le Kit QIAamp Viral RNA Mini Kit (Qiagen). La détection du VFVR a été réalisée par la technique de qRT-PCR à l’aide d’amorces et de sondes spécifiques.

**Résultats:**

Au total, 2 487 insectes (1 978 moustiques, 509 phlébotomes et 251 culicoïdes) ont été identifiés et repartis en trois familles (Culicidae, Psychodidae et Ceratopogonidae). La famille des Culicidae, composée entre autres du genre *Culex* a été la plus abondante, avec une prédominance de l’espèce *Cx. pipiens* (31,88 %; n = 793), suivie de *Mansonia* sp. (21,51 %; n = 535), *Anopheles gambiae* s.l. (8,44 %; n = 210), *An. pharoensis* (0,72 %; n = 18), *An. rufipes* (0,48 %; n = 12), *Cx. quinquefasciatus* (6,39 %; n = 159), celle des Psychodidae avec les phlébotomes (20,46 %; n = 509), et enfin celle des Ceratopogonidae avec le genre *Culicoides* (10,09 %; n = 251). La qRT-PCR réalisée sur un échantillon de 96 moustiques s’est révélée positive au VFVR chez un *Cx. pipiens.*

**Conclusion:**

Cette étude révèle pour la première fois la circulation du virus de la FVR chez *Cx. pipiens* au Niger et décrit ainsi le possible rôle vectoriel de cette espèce dans la transmission de la FVR. Cependant, d’autres investigations doivent être menées afin d’identifier les déterminants biologiques et écologiques qui supportent le maintien du virus dans cette zone afin d’orienter les interventions de lutte.

## Abstract (en)

**Background:**

The Rift Valley Fever (RVF) is an arbovirus disease responsible of regular epizootics and epidemics in sub-Saharan Africa and Arabian Peninsula. In 2016, Niger experienced its first outbreak of RVF in Tahoua region, which resulted in high consequences in animal and human health. The aim of this study was to investigate on the RVFV circulation among potential vectors of the disease.

**Methods:**

This was a cross-sectional survey carried out in Tahoua and Agadez regions in August 2021. Adult mosquitoes were collected by using the morning spray in human dwellings and the CDC light trap methods. After morphological identification, viral RNA was extracted. The RNA was extracted by using QIAamp Viral RNA Mini Kit (Qiagen). The RVFV detection was performed by using the qRT-PCR method.

**Results:**

A total of 2487 insects (1978 mosquitoes, 509 sandflies and 251 biting midges) were identified and divided into three families (Culicidae, Psychodidae and Ceratopogonidae). The Culicidae family composed of the *Culex* genus being the most abundant with a predominance of *Cx.pipiens* (31.88%; n = 793) followed by *Mansonia sp* (21.51%; n = 535), *Anophelesgambiae* s.l. (8.44%; n = 210), *An. pharoensis* (0.72%; n = 18), *An. rufipes* (0.48%; n = 12), *Cx. quinquefasciatus* (6.39%; n = 159), the Psychodidae with sandflies (20.46%; n = 509), and the Ceratopogonidae with *Culicoides* genus (10.09%; n = 251). The qRT-PCR carried out on a sample of mosquitoes (N = 96) highlighted that one individual of *Cx.pipiens* was found positive to RVFV. This specimen was from Tassara locality (Tahoua) and collected by CDC Light Trap method.

**Conclusion:**

This study reveals for the first time the circulation of RVFV among *Cx.pipiens* in Niger and highlights the possible vectorial role of this vector in the disease transmission. Further investigations should be carried out to identify the biological and ecological determinants that support the maintenance of the virus in this area in order to guide control interventions.

## Introduction

La fièvre de la vallée du Rift (FVR) est une zoonose virale transmise aux humains et aux ruminants par la piqûre des moustiques [9]. L’humain peut s’infecter aussi en manipulant les tissus et les liquides biologiques des animaux infectés [9, 18]. Elle est endémique en Afrique et a sévi dans la péninsule arabique, avec un impact significatif sur l’économie des populations [9, 18]. L’impact sur l’économie est dû aux avortements et aux cas de décès chez le bétail lors des épizooties qui réduisent ainsi la production, mais aussi à l’embargo sur l’exportation de têtes de bétail et de leurs produits durant ces périodes [18].

La FVR est apparue pour la première fois au Kenya en 1931 [3], puis le virus de la FVR s’est étendu progressivement à l’ensemble du continent africain, mais aussi au Moyen-Orient où il a provoqué de nombreuses épizooties et épidémies [18]. Cette zoonose, due à un virus appartenant au genre *Phlebovirus,* est transmise par des arthropodes, en particulier des insectes des genres *Aedes* et *Culex*, mais aussi *Anopheles, Eretmapodites, Mansonia* [9]. Les épizooties de FVR sont généralement déclenchées après d’intenses pluies qui engendrent la pullulation des vecteurs. Les épizooties sont suivies généralement de l’expansion du virus aux humains [16].

En 2016, le Niger a enregistré pour la première fois une épizoo-épidémie de la FVR à Tahoua [8], survenue suite à de fortes précipitations [11]. Chez les humains, il y a eu 399 cas avec 33 décès, soit un taux apparent de létalité de 8 % [8]. Chez les animaux, sur 2 383 cas suspectés, il y a eu 1 276 cas d’avortements et 476 morts [10]. Les facteurs de risque associés chez les humains au niveau du district de Tchintabaraden, l’épicentre ayant enregistré le premier cas humain, sont la consommation de lait, de produits laitiers et de viande provenant d’animaux malades [2]. Une étude récente conduite sur les mêmes sites de cette présente étude, en plus d’autres localités de Tahoua, révèle une séroprévalence globale d’anticorps anti-VFVR chez les ruminants domestiques de 30,62 % chez les bovins, 40,90 % chez les ovins et 37,21 % chez les caprins [7].

Malgré l’épizoo-épidémie de la FVR au Niger et les informations récentes sur la séroprévalence chez les ruminants domestiques, aucune information n’est disponible sur l’incrimination d’un vecteur dans la transmission. Ceci rend hypothétique les connaissances sur le cycle de transmission vectorielle avec pour conséquence un manque d’information pour la mise en œuvre d’une stratégie de lutte appropriée. C’est dans ce contexte que cette étude a été conduite pour analyser les risques entomologiques de réémergence de la fièvre de la vallée du Rift. Cela à travers la recherche du virus chez les vecteurs potentiels à Tahoua (Abalak, Tassara, et Tchintabaraden) et à Agadez (Ingall).

## Matériels et méthodes

### Type et période d’étude

C’est une étude transversale non répétée qui s’est déroulée du 5 au 18 août 2021. Elle a permis d’échantillonner des moustiques adultes par pulvérisations et par pièges lumineux CDC.

### Sites d’étude

L’étude a été conduite dans la région de Tahoua, où la première épizoo-épidémie de la FVR est apparue dans le pays en 2016, et dans la région d’Agadez. À Tahoua, 3 sites ont été choisis : Tassara (10°22’352” nord et 6°05’957” ouest), Tchintabaraden (15°53’587” nord et 5°47’550” ouest), Abalak (15°27’405” nord et 6°16’226” ouest) et un site à Agadez : Ingall (16°47’209” nord et 6°56’117” ouest).

**Figure 1:**
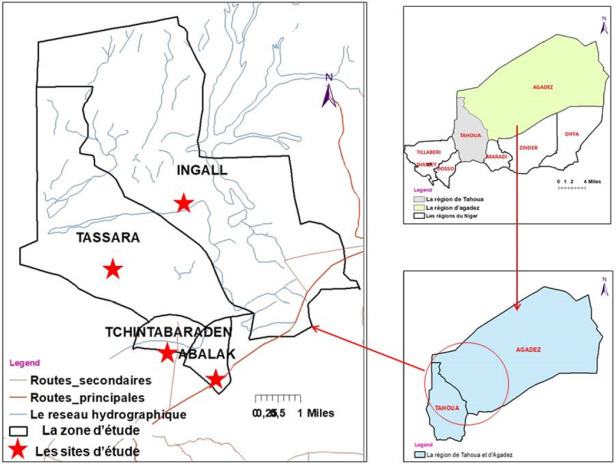
Localisation des zones de capture des moustiques dans les Région de Tahoua et d’Agadez Location of mosquito capture zones in the Tahoua and Agadez Regions

Le choix des sites de prélèvement a tenu compte des sites ayant enregistré le premier cas humain de FVR (Tchintabaraden), des sites associés à des mortalités et des avortements d’animaux (Abalak et Tassara) et a inclus enfin le département d’Ingall qui reçoit chaque année l’évènement culturel annuel « la cure salée » rassemblant des milliers d’animaux provenant de l’intérieur du Niger et des pays frontaliers (Fig. 1).

Ces sites sont caractérisés par des températures qui varient entre 25 °C et 40 °C selon la période de l’année. Ils sont de type sahélo-saharien avec une saison humide où le nombre de jours pluvieux varie de 18 à 26 jours avec des moyennes de 22 jours. La saison sèche dure neuf mois, d’octobre à juin. La saison froide se situe entre décembre à février. Ces sites constituent les zones pastorales par excellence du pays.

### Collecte des insectes, vecteurs potentiels de la FVR

Des captures de moustiques adultes ont été faites de deux manières : i) récolte de la faune résiduelle par pulvérisation matinale dans les habitations humaines (n = 40) et ii) capture à l’aide de pièges lumineux nocturne dans les cours des maisons (n = 8).

La pulvérisation matinale consiste à effectuer une pulvérisation spatiale de pyréthrine pour paralyser les moustiques se reposant à l’intérieur des habitations et à les ramasser sur des draps blancs étendus au sol ou sur les autres surfaces planes de l’habitation.

Les pièges lumineux ou CDC miniature lighttrap sont des pièges construits pour fonctionner de nuit dans le but de capturer les insectes nocturnes. Ils sont alimentés par une batterie (12 V). Ils sont placés le soir, à la tombée de la nuit et retirés le matin. Pour notre étude, ces pièges ont été placés à une hauteur de 60 cm environ au-dessus du sol, aux alentours du lieu de repos des animaux. La récolte des pots de capture des moustiques a été faite au lever du soleil.

### Identification et mise en lots des moustiques capturés

Avant l’identification, les insectes capturés ont été tués en les plaçant à -20 °C dans des thermos contenant des blocs réfrigérants pendant au moins 30 minutes, puis triés directement, tout en maintenant la chaîne de froid, afin d’éviter la dégradation des virus recherchés. À l’aide d’une loupe binoculaire, les espèces ont été identifiées à l’aide des clefs taxonomiques d’identification de Gillies et Coetzee 1987 [6] pour les espèces du genre *Anopheles,* la méthode de Mattingly [14] pour l’identification *dAedes aegypti* et celle d’Edwards [5] pour les autres Culicinae. Pour la distinction entre *Cx. pipiens* et *Cx. quinquefasciatus,* nous nous sommes basés sur les principales caractéristiques suivantes : *Cx. pipiens* est caractérisé par un « scutum avec des écailles brun-rougeâtres », alors que *Cx. quinquefasciatus*, est caractérisé par un « scutum avec des écailles chamois ». De plus, au niveau des ailes, la nervure R2+3 est plus longue chez *Cx. quinquefasciatus* que chez *Cx. pipiens.* Une fois le tri effectué, les moustiques femelles ont été regroupés en lots de un à cinq par genre et par espèce dans des tubes Eppendorf de 1,5 ml dans de l’éthanol absolu. Ensuite, les échantillons ont été immédiatement placés au frais dans des thermos contenant des blocs réfrigérants. Le transport des échantillons des localités de collecte jusqu’à Niamey a duré 24 heures au maximum. Une fois de retour au laboratoire, ces lots ont été stockés dans un congélateur à -80 °C avant les analyses de biologie moléculaire.

### Recherche du virus de la FVR par qRT-PCR

La recherche de virus a été intégralement exécutée dans le laboratoire de virologie du Centre de recherche médicale et sanitaire, qui est aussi le laboratoire national de référence pour les fièvres hémorragiques virales et autres zoonoses. La détection du VFVR a été réalisée par la technique de qRT-PCR.

Les échantillons de moustiques ont été préalablement broyés dans 500 pl de solution saline tamponnée au phosphate (PBS) à pH = 7. Les ARN des virus ont été extraits manuellement à l’aide du kit QIAamp Viral RNA Mini de

QIAGEN (Réf 52906) et l’amplification de l’ARN s’est faite sur un thermocycleur en temps réel de type QuantStudio5 d’Applied Biosystem à l’aide de l’enzyme QuantiTect Probe kit (Qiagen). La détection du VFVR a été réalisée en utilisant des amorces et sondes spécifiques (forward TGCCACGAGTYAGAGCCA, reverse TTGAACAGTGGGTCCGAGA et probe 6FAM-TCCTTCTCCCAgTCAgCCCCA C-BHQ1) telles que décrites précédemment par Bob *et al.* [1].

La détection de l’ADN cible se traduit par une courbe exponentielle. La détection de la séquence cible avant le 38^e^ cycle (Ct ≤38) confirme sa présence dans l’échantillon et donc, sa positivité.

## Résultats

### Composition des espèces capturées

Les deux méthodes de capture ont permis de recenser 2 487 insectes appartenant à trois familles : Culicidae, Psychodidae et Ceratopogonidae. Sur l’ensemble des 4 sites, 8 jours ont été consacrés à un total de 40 pulvérisations intra-domiciliaires et 8 nuits à un total de 8 piégeages CDC LT. Les pièges CDC ont été placés dans la cour des maisons prospectées à côté des enclos des animaux (incluant des ovins, des caprins et des bovins).

De manière générale, dans la famille des Culicidae, les espèces du genre *Culex* ont été les plus abondantes avec une prédominance de l’espèce *Cx. pipiens* (31,88 %; n = 793), suivi du genre *Mansonia* (21,51 %; n = 535), d’An. *gambiae* s.l. (8,44 %; n = 210), *An. pharoensis* (0,72 %; n = 18), *An. rufipes* (0,48 %; n = 12), *Cx. quinquefasciatus* (6,39 %; n = 159), des Psychodidae (20,46 %; n = 509) et des Ceratopogonidae avec le genre *Culicoides* (10,09 %; n = 251) (Tableau I). *Cx. pipiens* était l’espèce la plus abondante observée à Ingall (86,29 %; n = 248), à Abalak (33,20 %; n = 255), à Tchintabaraden (22,51 %; n = 138) et à Tassara (32,27 %; n = 152). Le genre *Mansonia* a majoritairement été observé à Abalak (28,41 %), Tchintabaraden (25,42 %) et Tassara (23,73 %). Les phlébotomes étaient abondants sur les sites d’Abalak (40,66 %), d’Ingall (24,95 %) et de Tassara (22,59 %).

**Tableau I:** Nombre de spécimens par localité et technique de capture Number of specimens by locality and capture technique

Site	Ingall		Abalak		Tchintabaraden		Tassara		Total
Espèces capturées	Pulvérisation (%)	CDC-LT (%)	Pulvérisation (%)	CDC-LT (%)	Pulvérisation (%)	CDC-LT (%)	Pulvérisation (%)	CDC-LT (%)	
*An. gambiae* s.l	0 (0)	0 (0)	49 (9)	0 (0)	59 (17)	102 (39)	0 (0)	0 (0)	**210**
*An. rufipes*	0 (0)	0 (0)	3 (1)	0 (0)	2 (1)	7 (3)	0 (0)	0 (0)	**12**
*An. pharoensis*	0 (0)	0 (0)	10 (2)	0 (0)	5 (1)	3 (1)	0 (0)	0 (0)	**18**
*Cx. pipiens*	152 (46)	96 (31)	202 (37)	53 (24)	80 (23)	58 (22)	52 (26)	100 (37)	**793**
*Cx. quinquefasciatus*	77 (23)	2 (1)	29 (5)	10 (5)	17 (5)	2 (1)	0 (0)	22 (8)	**159**
Phlébotomes	55 (17)	72 (24)	123 (23)	84 (38)	60 (17)	0 (0)	51 (25)	64 (24)	**509**
*Culicoides* spp.	0 (0)	61 (20)	0 (0)	53 (24)	82 (23)	0 (0)	55 (27)	0 (0)	**251**
*Mansonia* spp.	45 (14)	75 (25)	130 (24)	22 (10)	48 (14)	88 (34)	45 (22)	82 (31)	**535**
**Total**	**329 (100)**	**306 (100)**	**546 (100)**	**222 (100)**	**353 (100)**	**260 (100)**	**203 (100)**	**268 (100)**	**2487**

### Composition des espèces collectées par pulvérisation intra-domiciliaire

**Figure 2:**
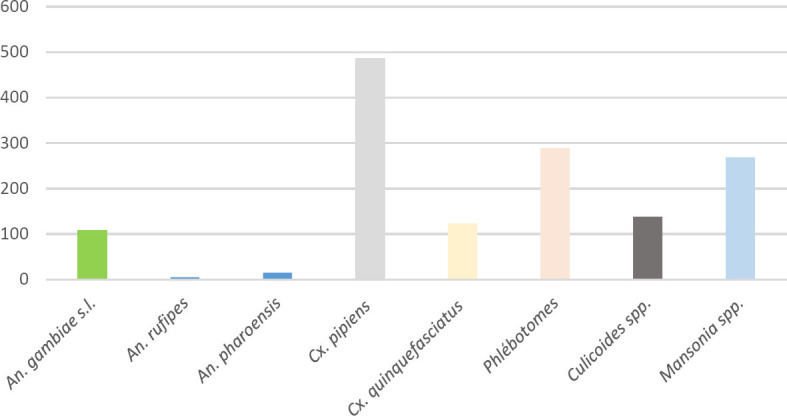
Répartition des insectes collectés par pulvérisation intra-domiciliaire Number of individuals collected by the Pyrethrum Spray Catch method

Au total, 1 431 individus ont été collectés par pulvérisation intra-domiciliaire sur une durée de 8 jours à raison de deux jours/site, dans 40 chambres, à raison de 10 chambres par site. *Cx. pipiens* était majoritaire avec 33,96 % (n = 486), suivi des phlébotomes 20,19 % (n = 289) et de *Mansonia* 18,72 % (n = 268). Le reste des diptères a été collecté dans les proportions suivantes : *Culicoides* spp : 9,57 % (n = 137), *Cx. quinquefasciatus* : 8,59 % (n = 123), *An. gambiae* s.l : 7,54 %, (n = 108), *An. pharoensis* : 1,04 % (n = 15) et *An. rufipes :* 0,35 % (n = 5) (Fig. 2).

### Composition des espèces collectées par piège lumineux CDC

**Figure 3:**
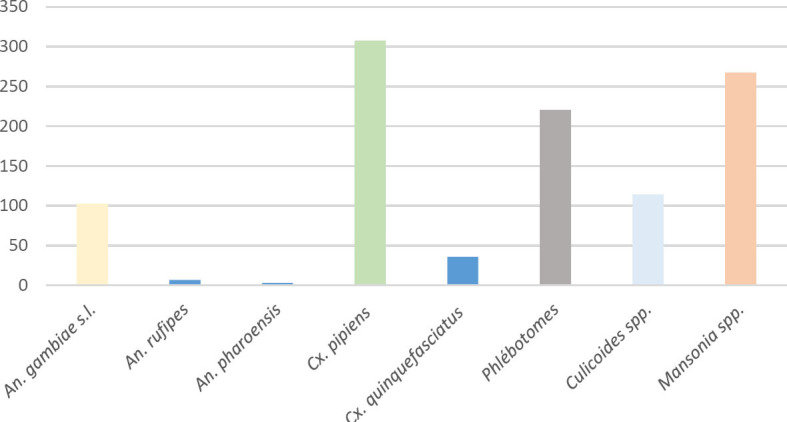
Répartition des insectes collectés par la méthode du piège lumineux (CDC LT) Number of individuals collected by the CDC Light Trap method

Avec la méthode au piège lumineux (CDC LT), 1 562 moustiques ont été collectés sur l’ensemble des quatre sites sur une durée de huit nuits à raison de deux nuits par site. Deux maisons par site ont été concernées, soit un total de 8 maisons sur l’ensemble des sites. La capture a rapporté 19,65 % de *Cx. pipiens* (n = 307), 17,09 % de *Mansonia* spp. (n = 267), 14,08 % de phlébotomes (n = 220), 7,29 % de *Culicoides* spp. (n = 114), 6,53 % *dAn. gambiae* s.l. (n = 102), 0,44 % d’An. *rufipes* (n = 7) et 0,19 % *d’An. pharoensis* (n = 3) (Fig. 3).

### Résultats virologiques chez les espèces de moustique par qRT-PCR

L’analyse virologique menée sur un échantillon randomisé couvrant des moustiques femelles capturés dans les différents sites (n = 96) a mis en évidence la circulation du VFVR chez un *Cx. pipiens*, soit un taux de positivité de 1 % (Tableau II). En effet, un échantillon a été testé positif au VFVR avec un seuil de détection (Ct) de 33,47. Les 96 individus analysés étaient tous gravides et aucun n’était gorgé. Cet échantillon positif a été collecté avec un piège lumineux CDC et provient de la localité Tassara, dans une cour de maison avec un enclos de ruminants domestiques.

**Tableau II:** Statut d’infectivité des espèces d’insectes au virus de la fièvre de la vallée du Rift Insect’s species infectivity status to the Rift Valley Fever Virus

Site	Nombre	Espèce	Résultat RT- qPCR
Tassara	16	*Cx. pipiens*	1 Positif sur 16
	5	*Cx. quinquefasciatus*	Négatif
	1	*Phlébotome* sp.	Négatif
	5	*Culicoides* sp.	Négatif
Abalak	5	*Cx. pipiens*	Négatif
	8	*Cx. quinquefasciatus*	Négatif
	9	Phlébotomes	Négatif
Ingall	10	*Cx. pipiens*	Négatif
	9	*Cx. quinquefasciatus*	Négatif
Tchintabaraden	3	*Phlébotomes*	Négatif
	6	*An. gambiae* s.l.	Négatif
	11	*Culicoides* sp.	Négatif
	2	*Cx. pipiens*	Négatif
	6	*Cx. quinquefasciatus*	Négatif
**Total**	**96**		**96**

## Discussion

Cette étude transversale a pour objectif d’identifier les probables vecteurs impliqués dans la transmission de la fièvre de la vallée du Rift dans les localités de Tchintabaraden, Tassara, anciens épicentres de l’épizoo-épidémie de 2016, mais aussi à Abalak et à Ingall, localités frontalières aux deux précédentes. Elle analyse aussi le risque de réémergence de la FVR au Niger cinq ans après la première épidémie.

Le diagnostic du VFVR a été fait par qRTPCR. Les résultats de cette étude ont rapporté que trois familles d’insectes hématophages ont été recensées : Culicidae, Psychodidae et Ceratopogonidae.

Beaucoup d’espèces de moustiques sont connues pour être des vecteurs compétents du VFVR, notamment les espèces des genres *Culex, Aedes, Anopheles, Eretmapodites* et *Mansonia*. Il est à noter dans cette étude que deux espèces de *Culex* ont été identifiées, à savoir *Cx. quinquefasciatus,* et *Cx. pipiens* majoritairement. Il faut dire que la distinction morphologique entre ces deux espèces n’est pas toujours aisée et peut porter à confusion. Mais nous nous sommes basés sur les caractéristiques des scutums pour faire la distinction comme évoqué plus haut. La présence des espèces de *Culex* en abondance a déjà été démontrée par l’investigation entomologique conduite lors de l’épidémie de 2016, notamment *Cx. quinquefasciatus, Cx. perexiguus, Cx. etbiopicus* et *Cx. antennatus* à Tchintabaraden et à Tassara. Les 34 pools de moustiques alors testés au VFVR ont été négatifs à l’issue des analyses [12].

Aucun moustique du genre *Aedes* n’a été collecté durant la période de l’étude. Cela pourrait s’expliquer par le fait qu’il existe des espèces d’*Aedes* qui ne produiraient qu’une seule génération par saison des pluies : leur population explose en début de saison et disparaît ensuite assez rapidement [19]. Les méthodes de collecte utilisées au cours de cette étude pourraient ne pas avoir été adaptées aux *Aedes*. Des résultats similaires ont été retrouvés pendant l’investigation de l’épidémie de 2016 où une seule espèce d’Aedes a été collectée, *Ae. dalzieli,* en nombre très bas (n = 1) à Tahoua [12].

À l’issue de cette étude, les résultats virologiques ont mis en évidence la présence du VFVR chez *Cx. pipiens* à Tassara. Cette étude n’a pas été couplée malheureusement à une étude de séroprévalence/clinique de la FVR chez les humains permettant de faire le lien de transmission avec la circulation du virus chez les moustiques. Même si le taux de positivité de 1 % sur un nombre relativement faible (n = 96) de moustiques testés laisse penser à un résultat faussement positif, les données récentes de séroprévalence de la FVR chez les ruminants domestiques à Tassara font penser le contraire. Une étude sérologique récente, menée dans cette même localité de Tassara chez les ruminants domestiques, révèle une prévalence élevée d’anticorps anti VFVR chez 50 % des bovins et des prévalences relativement faibles de 13 % et 4 % chez les ovins et caprins respectivement [7]. Cela est une preuve apparente de la circulation du VFVR chez les animaux domestiques à Tassara et dans le reste des localités, notamment à Tchintabaraden, Abalak où la circulation du virus a été détectée par Hama *et al.* [7]. La probable transmission humaine à bas-bruit est plausible dans ce contexte, mais avec l’absence d’une surveillance épidémiologique de routine, cette transmission est très probablement sousdiagnostiquée. Dans ce même ordre d’idée, on peut constater que, depuis l’épidémie de 2016, aucune étude de séroprévalence ou clinique n’a été visiblement conduite chez les humains pour évaluer la circulation du virus. Ce manque d’information peut constituer un biais dans l’évaluation du risque de réémergence de la maladie.

Il est urgent de produire des données de séroprévalence humaine afin de compléter les informations entomologiques et vétérinaires déjà disponibles. Pour l’instant, on peut estimer que les ruminants domestiques constituent des réservoirs biologiques potentiels concourant ainsi au maintien du VFVR dans ces localités [7].

Les espèces de *Culex* font partie des espèces chez qui le VFVR a été isolé un peu partout en Afrique [9]; elles sont compétentes pour transmettre le virus, comme *Cx. quinquefasciatus* et *Cx. poicilipes* au Sénégal [15]. Au Sénégal toujours, l’étude de Marrama *et al.* [13] a décrit le rôle de *Cx. quinquefasciatus* dans la transmission domestique du VFVR à Diawara et Diallo *et al.* [4] ont aussi identifié *Cx. poicilipes* comme vecteur de la FVR lors des épidémies de 1998-1999. Une autre étude similaire réalisée au Soudan [17], a incriminé l’espèce *Cx. pipiens* dans la transmission du virus en plus des moustiques du genre *Anopheles* au cours de l’épidémie de 2007. Même si leurs œufs ne survivent pas à la sécheresse, les espèces de *Culex* sont capables de coloniser progressivement les mares temporaires au cours de la saison des pluies, à partir de points d’eau permanents [16]. Ces espèces sont incriminées dans la phase d’amplification épidémique au cours de la seconde partie de la saison des pluies, car elles peuvent être extrêmement abondantes et possèdent de bonnes capacités vectorielles. Ce résultat montre la persistance et le maintien de la circulation du VFVR à Tassara, l’un des épicentres de l’épidémie de 2016. Cinq ans après, le risque d’une réémergence semble réel dans cette localité, surtout si les conditions pluviométriques favorables s’y ajoutent. Ce qui donne l’idée de considérer ces localités de la région de Tahoua (Tassara et Tchintabaraden) comme un « hotspot » du VFVR au Niger : la surveillance devrait y être active afin d’éviter la résurgence de la maladie. D’autres investigations doivent être menées afin i) d’identifier les déterminants (écologiques et biologiques) qui supportent le maintien du virus aussi bien en saison pluvieuse qu’au cours du reste de l’année, ii) d’évaluer les compétences vectorielles au VFVR des vecteurs rencontrés dans cette étude. Cela permettrait de mener par la suite des interventions de contrôle et de lutte fondées sur des preuves afin de pouvoir sauver des vies et sauvegarder l’économie des populations.

À court terme, des mesures telles que la lutte antivectorielle par pulvérisation intradomiciliaire d’insecticide à effet rémanent, l’information et la sensibilisation de la population sur le risque d’une réémergence de la FVR aideraient pour contrôler cette zoonose.

## Conclusion

L’enquête sur l’évaluation du risque de réémergence de la fièvre de la vallée du Rift à Tassara, Tchintabaraden, Abalak et Ingall a permis de dresser un inventaire des populations de moustiques et phlébotomes qui colonisent ces zones : *An. gambiae* s.l., *An. rufipes, An. pharoensis, Cx. pipiens, Cx. quinquefasciatus, Culicoides* spp. et *Mansonia* spp.

La mise en évidence du VFVR chez l’espèce *Cx. pipiens* par la qRT-PCR à Tassara, un des épicentres de l’épidémie de 2016, montre qu’il faut considérer cette zone de Tahoua comme une zone où la réémergence de la FVR est plus probable, surtout avec le concours des paramètres pluviométriques et l’intense activité pastorale. Ces localités doivent être priorisées lors des interventions de lutte antivectorielle contre la FVR et lors des campagnes vaccinales. Des études d’évaluation des compétences vectorielles au VFVR devraient être menées sur ces vecteurs recensés à Tassara et à Tchintabaraden, épicentres de l’épidémie passée, afin de mettre en œuvre une stratégie de lutte antivectorielle fondée sur des preuves. Aussi, à titre préventif, les campagnes de vaccination du bétail doivent être régulières et maintenues de façon durable.

## Remerciements

Nous adressons nos remerciements aux institutions qui nous ont soutenus dans la réalisation de ce travail : ministère de la Santé Publique et des Affaires Sociales, Projet REDISSE III, autorités locales et administratives.

## Financement

Les investigations de terrain ont été financées par le projet REDISSE III du Niger avec le soutien du Laboratoire central de l’élevage, du CERMES et de l’ENSP de Zinder.

## Contributions des auteurs

IS, AL, MAA, ZN ont conçu le travail, collecté les données et rédigé le premier draft. IML, ZN et MAA ont participé à l’analyse des données et à la relecture du manuscrit. Les données ont été collectée sous la supervision des auteurs IML et BF qui ont en même temps assuré la coordination du travail de toute l’équipe. Tous les auteurs ont lu et approuvé la version finale du manuscrit.

## Conflit d’intérêts

Les auteurs déclarent ne pas avoir de conflit d’intérêts.
